# O03 Antimicrobial pharmacist led penicillin allergy de-labelling (PADL) is safe and effective at removing low risk penA records

**DOI:** 10.1093/jacamr/dlaf118.003

**Published:** 2025-07-14

**Authors:** Daniel Hearsey, Jonathan Sandoe, Sarah Tonkin-Crine, Neil Powell

**Affiliations:** Pharmacy Department, Royal Cornwall Hospital Trust, Truro, UK; Leeds Institute of Medical Research, University of Leeds, Leeds, UK; Nuffield Department of Primary Care Health Sciences, University of Oxford, Oxford, UK; Pharmacy Department, Royal Cornwall Hospital Trust, Truro, UK

## Abstract

**Objectives:**

To implement a penicillin allergy de-labelling (PADL) intervention in adult medical and surgical specialties, delivered by both the wider clinical teams and antimicrobial stewardship (AMS) pharmacists.

**Methods:**

The core (PADL) implementation team were two antimicrobial stewardship (AMS) pharmacists. Hospital PADL guidelines and toolkit were approved by hospital management. Pharmacy technicians, ward pharmacists and Foundation Year doctors received PADL training. Senior clinicians and senior nurses were briefed on PADL at specialty meetings. A web-based surveillance report identified inpatients with a penicillin allergy (penA) record prescribed antibiotics. AMS pharmacists used the surveillance report to prioritize patients for PADL and championed PADL in ward areas between 10 June 2024 and 10 December 2024.

**Results:**

A total of 735 patients with a penicillin allergy (penA) record and prescribed any antibiotic had a structured penA history documented in the electronic prescribing system. Of these, 549 (74.7%) had a penicillin allergy assessment. After removing 52 patients who had opted out of research, we report on 497 (68%) remaining patients. In total, 136 (27%) of the penicillin allergy histories were taken by ward pharmacy technicians, 134 (27%) by ward pharmacists, 21 (4%) by ward doctors, and 206 (41%) by the AMS team (AMS pharmacists, Foundation Year doctors on medical microbiology rotation and AMS pharmacy technician). Of the 497 risk-assessed patients, 186 (37%) were both low risk and consented to be de-labelled either via direct de-label or (DDL) or direct oral challenge (DOC); 58/186 (31%) DOC and 128/186 (69%) direct de-label (DDL). Of these, 56/58 (97%) and 126/128 (98%) were successfully de-labelled via DOC and DDL, respectively. Patients were de-labelled via either by DOC or DDL by ward pharmacy technicians (1; 0.5%), ward pharmacists (24;13%), ward doctors (31; 17%) and the AMS team (130; 70%). All 58 (100%) DOC patients were exposed to amoxicillin during the DOC test with 44/58 (76%) going on to receive a course of penicillin post DOC. 75/128 (59%) DDLs were exposed to a penicillin antibiotic during their stay. There was no evidence of harm in the medical notes review for 172 (96.6%) tested / de-labelled patients (DOC 52 / DDL 120) with some evidence of potential harm in 6/178 (3.4%), all reactions reported were minor and most subjective reactions. Patient harm by PADL method: DOC 4/56 (7.1%) and DDL 2/122 (1.6%) e-notes unavailable for 2 DOC and 7 DDL patients and therefore unable to follow up these patients.

**Conclusions:**

AMS pharmacist led and delivered PADL working with both ward-based pharmacy and medical teams was safe and effective at removing low risk penA records. The impact of PADL on antibiotic use needs to be determined in our cohort.

